# Editorial: Accelerating cancer genomics research in Sub-Saharan Africa

**DOI:** 10.3389/fonc.2025.1675850

**Published:** 2025-09-26

**Authors:** Folakemi T. Odedina, Solomon O. Rotimi, Abdou Azaque Zoure

**Affiliations:** ^1^ Division of Hetalology/Oncology, Mayo Clinic, Jacksonville, FL, United States; ^2^ Prostate Cancer Transatlantic Consortium (CaPTC), Jacksonville, FL, United States; ^3^ Department of Biochemistry, Covenant University, Ota, Nigeria; ^4^ Département Biomédical et Santé Publique, Institut de Recherche en Sciences de la Santé (IRSS/CNRST), Ouagadougou, Burkina Faso

**Keywords:** cancer genomics, genomics research, sub-Saharan Africa, precision oncology, cancer research

The burden of cancer in Sub-Saharan Africa (SSA) is now approaching an epidemic proportion with concomitant poor outcomes and associated high mortality. SSA countries are projected to report the highest increase in cancer-associated mortality by 2040. In addition, this burden of cancer comes with huge economic losses. As a genomic disease, cancer presents with heterogenous phenotypes within different populations. However, the preponderance of cancer genomics research, cancer genetics research and clinical trials that have resulted in cancer control measures are conducted outside of SSA countries. This has limited the understanding of the biology of cancer among people of African ancestry, the utilization of precision oncology in SSA countries and the translational impact of new interventions in improving cancer outcomes in Sub-Saharan Africans. This Research Topic brings together five key manuscripts on: (1) the need to accelerate cancer genomics research to accelerate precision oncology; (2) current initiatives to foster genomic research in SSA; (3) profile of neoantigens in Kenyan breast cancer patients using genomic DNA and total RNA; (4) the future of collaborative precision oncology efforts tailored to SSA’s unique genomic and infrastructural landscape; and (5) the rapid evolution of non-invasive cancer biomarkers.

The review article by Ivanga et al. focused on “*Accelerating Cancer Genomics Research in SSA*”, highlighting both the challenges and initiatives aimed at advancing precision oncology in the region. The review emphasized the importance of cancer registries, biobanking and robust technical platforms as essential bedrock for cancer genomics research and for translating its finding into clinical practice. Amaeshi et al. advanced this conversation through a literature review on the “*Current Landscape of Cancer Genomics Research in SSA*”. The paper discussed the unique genomic characteristics of common cancers in Africa, such as the higher frequency of BRCA1 and BRCA2 mutations in breast cancer patients in certain African countries compared to other populations. It emphasized that cancer precision control remains stunted due to many challenges, including insufficient funding, inadequate infrastructure, and a limited pool of trained professionals. The potential solutions highlighted by Amaeshi et al. included establishing genomic research centers, enhancing training and capacity building, and increasing local funding through government investment and philanthropy. Amaeshi et al. and Ivanga et al. discussed the strides being made by initiatives such as the H3Africa Consortium, African BioGenome Project, and the Prostate Cancer Transatlantic Consortium (CaPTC).

Wagutu et al. concluded that “*Whole Exome-seq and RNA-seq Data Reveal Unique Neoantigen Profiles in Kenyan Breast Cancer Patients*”, exemplifying the importance of African cancer genomics research to advancing knowledge of carcinogenesis and addressing the global burden of cancer. The study profiled neoantigens in 23 Kenyan breast cancer patients using whole exome sequencing (WES) and RNA sequencing of paired tumor and adjacent non-cancerous tissues. The authors reported that an average of 1,465 neoantigens covering 10,260 genes were identified per patient. Out of the 58 COSMIC genes commonly mutated in breast cancer, 44 (76%) produced more than two neoantigens. Across all genes, 2,809 mutations were detected mutations, the majority of which were the missense type, most of which were substitutions of C>T. Overall, the study highlights the unique neoantigen profiles in Kenyan breast cancer patients, suggesting their potential as biomarkers for prognosis and in personalized immunotherapy.

Achieving the goals of accelerating precision oncology in Africa also hinges on ideas elaborated by Gueye et al. in their perspective article titled “*The Future of Collaborative Precision Oncology Approaches in Sub-Saharan Africa: Learnings from Around the Globe*”. They argue that the promise of technologies like comprehensive genomic profiling (CGP) can only be realized in SSA through strategic, collaborative consortia, as also stated by Ivanga et al.. The authors document global case studies—from the UK NHS’s 100,000 Genomes Project to Germany’s DKTK platform—and highlight how cross-border collaboration, regional capacity building, and data-sharing platforms can catalyze innovation in Africa. Their paper places a strong emphasis on genomic diversity and equity, noting the underrepresentation of African populations in major genomic datasets.

In the systematic review titled “*Emerging Biomarkers for Non-Invasive Diagnosis and Treatment of Cancer*”, Zakari et al. synthesized evidence from 45 studies exploring the burgeoning promise of non-invasive biomarkers—such as circulating tumor DNA (ctDNA), exosomes, and microRNAs—for cancer detection, monitoring, and therapy. By cataloging applications across various tumor types—including breast, prostate, lung, and colorectal—the review highlights how liquid biopsies and epigenetic markers offer alternatives to invasive tissue biopsies. The article systematically details the strengths and limitations of non-invasive methods and underscores their transformative potential in enhancing early detection, personalizing treatment, and improving outcomes, particularly in low-resource settings where repeated tissue access is impractical.

To address the burden of cancer in SSA countries, it is imperative to upscale the penetration of precision oncology in Africa by accelerating the inclusion of the SSA population in cancer genomics research. These Research Topic contributions signal a paradigm shift in oncology: from late-stage intervention to precision prevention and therapy; from siloed approaches to globally integrated, context-specific models. Importantly, these articles spotlight the necessity of aligning innovation with infrastructure and inclusivity—emphasizing that breakthroughs in cancer care will be most impactful when tailored to diverse populations and health systems.

Genomics research requires intensive resources and high-level expertise that are often beyond the limits of individual researchers or institutions, particularly in low-resource settings. This Research Topic serves as a call to action. It highlights both the technological possibilities, and the collaborative pathways needed to close global cancer equity gaps. While non-invasive biomarkers offer a scalable solution for earlier detection and monitoring, implementing these tools in SSA will require systemic investments, consortia-based collaboration, and policies that foreground African genomic inclusion. These articles lay the groundwork for such efforts, offering a scientific and strategic blueprint for accelerating precision oncology in the region.

